# Caries prevalence among children at public and private primary schools in Riyadh: a retrospective study

**DOI:** 10.1186/s12903-024-04570-6

**Published:** 2024-07-17

**Authors:** Budur Almutairi, Tasneem Rashed Adam, Rami Bustami

**Affiliations:** 1 Saudi Ministry of Health, Riyadh, Saudi Arabia; 2 College of Applied Medical Sciences, King Saud University, Riyadh, Saudi Arabia; 3 College of Business, Alfaisal University, Riyadh, Saudi Arabia

**Keywords:** Dental caries, Schoolchildren, Socioeconomic conditions, Prevalence

## Abstract

Dental caries is a global oral health issue, especially critical in children, affecting their growth, nutrition, and education due to school absences or distractions from dental pain. The aim of the study was to investigate the correlation between school types (indicative of socioeconomic conditions) and dental caries prevalence among primary school children in Riyadh, alongside assessing the overall caries prevalence among schoolchildren in Riyadh. Retrospective study on 28,343 first and fourth-grade students from 960 public and private schools in Riyadh, using data from the Saudi Ministry of Health (Feb-April 2019). Utilized the DMFT/dmft index for assessment and collected demographic data. Most of the schools were public (76.1%), private national (17.1%), and private international (6.8%). Overall, the mean DMFT index for permanent teeth and the dmft index for primary teeth were 1.78 and 1.94, respectively. 58% of school children had no dental caries, 25% had mild caries, and 17% had moderate to severe caries. Public school children showed a higher caries prevalence than private schools. Oral disease rates were higher in girls than in boys, and grade four students had a higher prevalence than grade one students. Saudi Arabia, a developing nation, faces challenges in addressing oral health, especially in public schools. Targeted initiatives are crucial for awareness, preventive measures, and meeting oral health needs.

## Introduction

Dental caries poses a global oral health challenge, causing pain and requiring costly treatment that can impact nutrition and overall well-being. Particularly concerning in children, dental caries not only weakens affected tooth structures even after treatment but also hinders growth and development. Children may experience poor development and nutrition when chewing is painful, leading to school absences or decreased concentration in older children. Additionally, young individuals may withdraw from social interactions because of discomfort or embarrassment about their teeth. The far-reaching effects of dental caries on physical, social, emotional, psychological and cognitive development have lasting implications for success and productivity throughout life [1, 2]. The prevalence of dental caries is increasing in developing countries for various reasons, including the intake of cariogenic foods, poor oral hygiene, socioeconomic status, health, age, access to health services and other lifestyle factors [1].

Numerous published studies have demonstrated the link between attending public school and socioeconomic conditions, highlighting distinct differences among students from various socioeconomic backgrounds [3–6]. Socioeconomic status plays an important role in school types, as parents with higher educational degrees and incomes are willing to enroll their children in private schools, aiming to provide them with a better environment and improve their academic performance [7]. In a 2022 study, Reima et al. reported that parents generally perceive English language education in public and Quranic schools to be lacking, and they favor international schools as a result. These parents believe that English is a more powerful and desirable language for their children in private schools [8]. This correlation extends to multiple facets, including academic performance, health outcomes, and behavioral patterns. The decision to opt for public or private schooling is significantly influenced by household socioeconomic status, with parents from higher socioeconomic backgrounds favoring private schools for their children. Key factors such as perceived education quality, resource accessibility, and the overall socioeconomic environment of the school play a pivotal role in shaping this parental decision [9].

Numerous studies in Saudi Arabia (SA) have examined the prevalence and severity of caries in children. According to the Ministry of Health’s (MOH) official website, approximately 96% of 6-year-olds and 93.7% of 12-year-olds experience dental caries. A review of 27 studies conducted between 1988 and 2010 revealed a high prevalence of dental caries in Saudi children, with approximately 80% for primary dentition (mean dmft score of 5.0) and approximately 70% for permanent dentition (mean DMFT score of 3.5) [10]. A literature review from 1999 to 2008 confirmed a consistent high prevalence, with a mean DMFT of 5.38 in primary teeth and a DMFT of 3.34 in permanent teeth [11]. A recent meta-analysis of different dental caries studies in different regions of SA determined the prevalence to be 80% [12]. The collective evidence from previous studies confirms the endemic nature of dental caries in the Middle Eastern population and signifies a burden on public health.

In 2019, the total number of schools across all regions in SA was approximately 38,000. Of these, 80.3% were public schools, which provide state-funded education. Private schools, accounting for 12.5% of all schools, are fee-paying institutions. Both public and national private schools deliver curricula approved by the Ministry of Education, while private international school offer international curricula such as American, British, or Indian, made up 6.2% of schools [13]. As of 2023, schools in the Riyadh region accounted for 24.1% of all schools in SA [14]. During the 2018–2019 academic year, across the SA, there were approximately 6.18 million students enrolled in elementary and high schools. Of these, about 82%, 11.1% and 5.9% attended public schools, private schools and international schools, respectively [13].

In SA, the Exploratory Examination of Students (EES) initiative is a health screening programme that has been implemented in schools across the nation. The programme’s objective is to detect health issues at an early stage, provide children with the necessary assistance, raise health awareness, and maintain a comprehensive health record for each student, regardless of whether they are Saudi citizens or residents. The programme is mandatory for all students and consists of four check-ups, which are conducted at different milestones, such as the first and fourth grades of primary school, the first grade of intermediate school, and the first grade of secondary school. The examination is a thorough process that covers a wide range of health concerns, including medical history, vaccination status, potential health issues such as obesity, tooth decay, vision and hearing problems, and mental health conditions such as depression, attention deficit hyperactivity disorder, and smoking habits. The program is carried out by a trained team comprising a doctor, a dentist, and a nurse. The doctor performs the physical examination, the dentist conducts the dental examination and makes referrals as required, and the nurse records the data. The programme aims to promote the health and well-being of students by integrating health services into the educational system [15, 16].

Several investigations have been conducted on the prevalence of caries in SA, with a focus on various social factors. However, the present study aimed to explore the correlation between school type (an indicator of socioeconomic conditions) and dental caries prevalence among primary school children in Riyadh. Additionally, this study sought to assess the overall prevalence of caries among schoolchildren in Riyadh.

## Materials and methods

This is a retrospective study utilising data from phase one of the EES initiative on elementary school students (in the first and fourth grades) enrolled in 960 public and private schools in Riyadh, SA. The Saudi MOH, which approved the use of its database, collected the data from the School Health Department between February and April 2019.

The Saudi MOH collected the data under a protocol designed for the EES initiative, which encompassed three key aspects relevant to this paper. Firstly, training the medical teams involved representatives from all health affairs regions during central workshops at the MOH in Riyadh. Subsequently, these representatives returned to their respective regions to train the medical teams locally. The trained dental team conducted dental screenings, involving a nurse recording basic data, a dentist performing examinations and providing health awareness when necessary, and a health counselor facilitating the process by coordinating with the school, collecting health history forms, preparing the examination area, and ensuring follow-up on referred cases.

Secondly, the target population comprised all students in first and fourth grades during the academic year 2018–2019. In phase one, the MOH aimed to target 50% of this total population. Finally, dental examinations were conducted in the school setting, in a quiet room away from students and noise. Children were asked to lie supine on a table, and their teeth were cleaned of food deposits and plaque using gauze. Dental examinations were performed using a dental mirror, probe, and appropriate lighting (blue-white spectrum, normal white lighting, or sufficient natural lighting), following the standard examination procedure, from the upper jaw to the lower jaw, and from right to left.

In addition to DMFT/dmft, several demographic data points were collected from students, including date of birth, gender, grade, and type of school (public, national private, or international private). DMFT/dmft levels were defined as follows: (1) no dental caries (DMFT/dmft = 0), mild (DMFT/dmf t = 1–3), and moderate to severe (DMFT//dmft = 4 or higher). The presence of dental caries was defined as a DMFT/dmft score of 1 or more.

The data were uploaded and saved in an appropriately designed Excel spreadsheet. Adhering to the National Data Management Office guidelines established by the Saudi Data and Artificial Intelligence Authority, these guidelines were followed throughout the data lifecycle, covering data collection, organization, cleaning, and validation. A specialized team supervised these processes to ensure data integrity and quality. Additionally, the data underwent thorough validation against the expected minimum and maximum values of DMFT/dmft. Any values falling outside this range were identified and flagged as implausible. A similar validation process was applied to demographic variables, where general frequency analyses were conducted to detect any potential anomalies.

Descriptive statistical analyses were performed on the data for the study sample. Continuous variables were summarized using the mean and standard deviation (SD), and proportions were used for categorical variables. The presence of carries was evaluated overall and compared between the two groups (private and public) using the chi-square test. Age was compared among children with caries vs. those with no caries using the t-test, if assumptions of normality and equal variances were met, otherwise the nonparametric Mann-Whitney U test was utilized. Comparisons of caries prevalence were made by gender and grade using the chi-square test. Also, a multivariate linear regression model was utilized to predict DMFT for each group. Adjustments were made for age, sex, and grade. P values < 0.05 were considered to indicate statistical significance. All the statistical analyses were performed using IBM SPSS 21.0 (Armonk, NY: IBM Corp).

## Results

A total of 28,343 schoolchildren were included. The descriptive statistics of the study sample are displayed in Table 1. The average age was 8.2 (SD = 2.6) years, and 56% of the participants were females. The distribution of school types was as follows: Public, 76.1%; private national, 17.1%; and private international, 6.8%. The data were balanced in terms of grade: 49.5% were grade one, and 50.5% were grade four. Overall, the mean DMFT index for permanent teeth and the dmft index for primary teeth were 1.78 and 1.94, respectively. 58% of school children had no dental caries, 25% had mild caries, and 17% had moderate to severe caries.

**Table 1:** Descriptive statistics of the study sample. *N* = 28,343

Category	*N* (%)
**Gender** n (%)	
Female	15,736 (55.5%)
Male	12,607 (44.5%)
**Age (years)** mean ± SD	8.2 ± 2.6
**School Type** n (%)	
Public	21,557 (76.1%)
Private national	4,856 (17.1%)
Private international	1930 (6.8%)
**Grade** n (%)	
First	14,028 (49.5%)
Fourth	14,315 (50.5%)
**DMFT/dmft index** mean	1.78/1.94
**DMFT Group** n (%)	
No caries	16,459 (58.1%)
Mild	6,941 (24.5%)
Moderate to severe	4,943 (17.4%)

The prevalence of caries was evaluated in 28,343 children, with 41.9% (11,884 children) found to have caries. The results of comparing caries prevalence by various demographic characteristics are presented in Table 2. The average age ± SD among children with caries was 8.2 ± 2.6 years, compared to 8.2 ± 2.6 years for those without caries (*p* = 0.15). The prevalence of caries was significantly higher among female children (42.6%) than male children (41.1%) (*p* = 0.014). Additionally, children enrolled in public schools had a significantly higher prevalence of caries (42.2%) compared to those enrolled in private international schools (37.6%) (*p* < 0.001). Public school children also had a marginally higher prevalence of caries than those enrolled in private national or international schools (*p* = 0.065). No significant differences were detected between first-grade and fourth-grade children in terms of caries prevalence (41.6% and 42.2%, respectively; *p* = 0.34).

**Table 2:** Descriptive statistics of the study sample by demographic and clinical factors. Total number of children = 28,343

Category	No caries (%)	Caries (%)	*p*-value*
**All children** n (%)	16,459 (58.1%)	11,884 (41.9%)	
**Gender** n (%)			0.014
Male	7,422 (58.9%)	5,185 (41.1%)	
Female	9,037 (57.4%)	6,699 (42.6%)	
**Age (years)** mean ± SD	8.2 ± 2.6	8.2 ± 2.6	0.15
**School Type** n (%)			< 0.001
Public	12,453 (57.8%)	9,104 (42.2%)	
Private national	2,802 (57.7%)	2,054 (42.3%)	
Private international	1,204 (62.4%)	726 (37.6%)	
Private (national and international)	4,006 (59.0%)	2,780 (41.0%)	
**Grade** n (%)			0.34
First	8,186 (58.4%)	5,842 (41.6%)	
Fourth	8,273 (57.8%)	6,042 (42.2%)	

The correlation between child’s DMFT score and school types, gender and grade were assessed using multivariate linear regression, as shown in Table 3. The results of the linear regression analysis indicate several significant associations with the DMFT score. Females had a significantly higher DMFT score compared to males (B = 0.58, 95% CI [0.53, 0.63], *p* < 0.001). Moreover, children attending public schools had a higher DMFT score compared to those attending private national or international schools (B = 0.45, 95% CI [0.38, 0.51], *p* < 0.001). Additionally, schoolchildren in the fourth grade had significantly greater DMFT scores than did those in the first grade (B = 0.16, 95% CI [0.1, 0.21], *p* < 0.001).

**Table 3:** Multivariate Linear Regression Model for DMFT Index. Total number of children = 28,343

Independent variable	Multivariate linear regression			
	B	95% CI	*p*-value	
**Intercept (DMFT Index)**	1.33	(1.26,1.40)		
**Gander**				
Male	-0.58	(-0.63,-0.53)	< 0.001	
Female	0.0	Ref.		
**School Type**				
Private	0.0	Ref.		
Public	0.45	(0.38,0.51)	< 0.001	
**Grade**				
First	0.0	Ref.		
Fourth	0.16	(0.1,0.21)	< 0.001	

*B* is the regression coefficient. CI: Confidence Interval. Ref.: Reference.

**Fig. 1:**
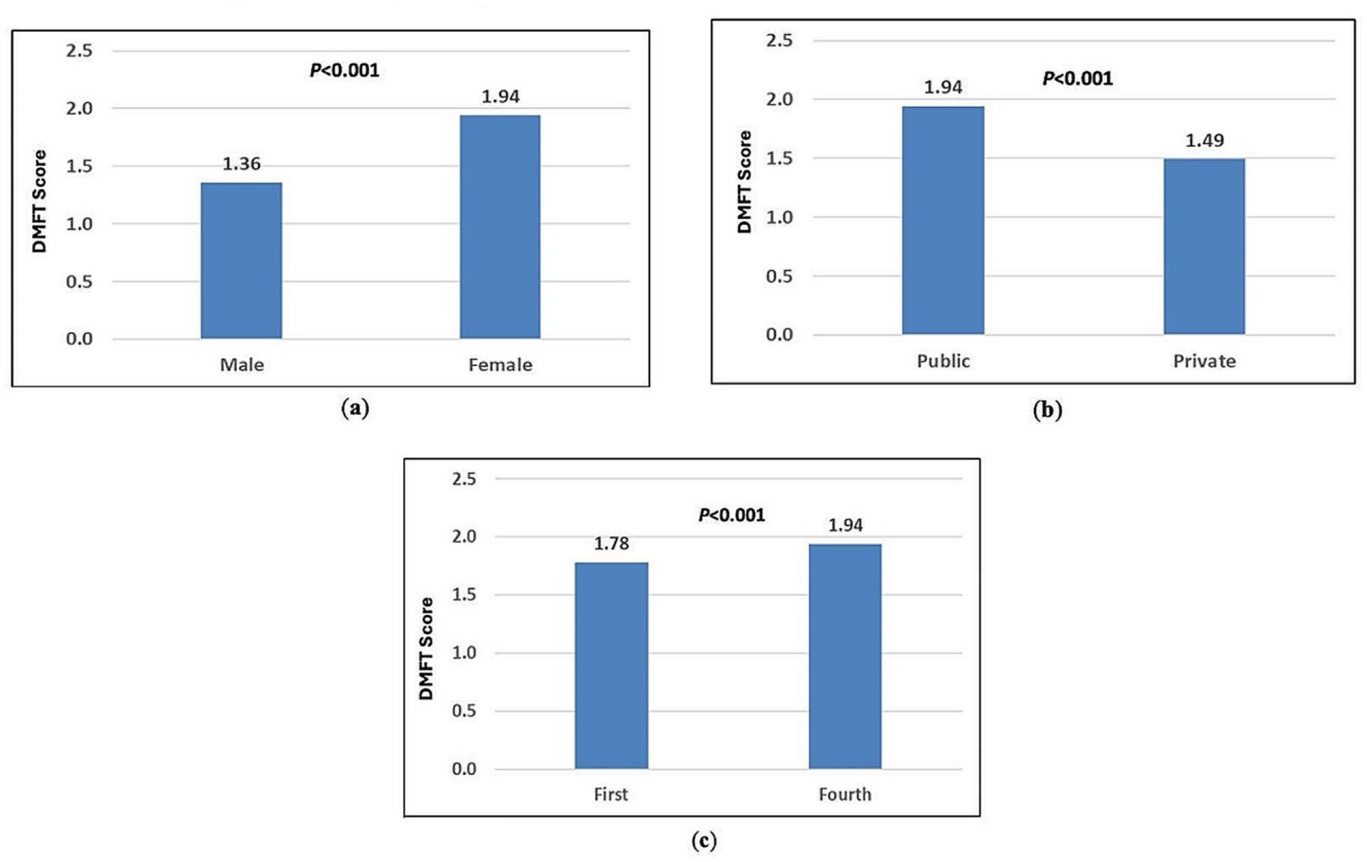
Based on the regression model in Table 3, for a child enrolled in a public school in fourth grade, (**a**) the average DMFT score was predicted by gender; (**b**) the average DMFT score was predicted by school type; and (**c**) the average DMFT score was predicted by grade

There were significant associations between caries experience and both school type and gender (p < 0.001). After adjusting for grade, the existence of differences in dental caries indicators was confirmed. Figure 1 shows that children enrolled in public schools had higher DMFT scores compared to those in private schools, with females in Grade 4 showing a DMFT of 1.94 versus 1.49 (p < 0.001). Additionally, the mean DMFT for children in public schools was higher for females than for males in Grade 4 (1.94 vs. 1.36, p < 0.001). This pattern was also observed across different grades. Furthermore, there was a gradient in the association between grade level and DMFT, with fourth graders in public schools having higher DMFT scores than first graders (1.94 vs. 1.78, p < 0.001) (Fig. 1).

## Discussion

According to the results of the current study, fourth-year students show a significantly greater prevalence of dental caries than first-year students. In agreement with our findings, a 2013 study reported that the prevalence of caries was 72.1% in primary teeth and 61.7% in permanent teeth [17]. Another study involving participants aged four to 14 years identified ten-year-olds as the most affected age group and four-year-olds as the least affected [1]. On the other hand, the opposite results were found in a study of 730 s- to fourth-grade children. Of the participants, 53.7% of Grade Two children and 14.4% of Grade Four children had caries, whereas Grade Four children had fewer caries than Grade Two children [18].

In our study, we found that the prevalence of caries was greater among children enrolled in public schools than among those enrolled in private schools. This finding aligns with similar findings in a study conducted in Brazil, which reported caries prevalence rates of 74.50% and 61.20% among public schoolchildren and private schoolchildren, respectively [19]. Comparable results were also noted in a study among 12- to 15-year-old schoolchildren in Jordan, where public schoolchildren exhibited higher prevalence rates than did their private school counterparts [20].

Moreover, a study involving 604 children revealed a significantly greater percentage of children in public schools with poor oral hygiene status. Generally, the prevalence of oral diseases is lower among children in private schools than among those in public schools [21]. The type of school was consistently linked to the oral health condition of children in various studies, such as one where the DMFT index was higher in children from public schools [22].

However, it is worth noting a contradictory finding in a separate study in which students in public schools had a significantly lower prevalence of caries (37.36%) than did those in private schools (47.96%) [23]. Therefore, in the present study, the majority of the study subjects from public schools belonged to the medium- to high-risk category, and private school subjects belonged to the low-risk category, which suggested that private school students have more opportunity to prevent dental caries than do public school students.

Public schools typically serve a higher proportion of low-income and less educated families compared to private schools [24, 25]. Public schools are state-funded institutions that provide education at no cost to families, which is why many low- to middle-income families choose these schools for their children. This economic factor is crucial, as several factors contribute to the observed differences in dental caries prevalence between students in public and private schools. This socioeconomic disparity is significant because lower income and education levels are often associated with limited access to dental care services, poor health literacy, poor oral hygiene, and less nutritious diets, all of which increase the risk of dental caries [26, 27]. Consequently, the type of school a student attends can serve as an effective indicator of socioeconomic status in dental health surveys [28], particularly in SA, where collecting detailed individual socioeconomic data can be impractical.

In our study, we observed that 42% of schoolchildren exhibited moderate to severe caries, while 58% had no dental caries. This finding diverges from several studies conducted in SA. In a systematic review comprising 27 published studies on caries among children in SA, the national prevalence of dental caries was estimated to be approximately 80% for primary dentition and approximately 70% for children’s permanent dentition [1]. Another meta-analysis revealed that the estimated prevalence of dental caries among children aged 5–7 years was 84%, while for those aged 12–15 years, it was 72% [29]. Additionally, another study reported a general prevalence of dental caries of almost 73%, with specific rates of approximately 78% for six- to nine-year-old and approximately 68% for ten- to 12-year-olds [11].

However, international studies on dental caries showed equivalent results to our study. A study conducted in Pakistan reported a caries incidence of 40.5% in preschool children aged three to five years [11]. In addition, the overall prevalence of caries in the Timor-Leste group was 64% [30]. China reported results comparable to the prevalence of dental caries. In one study, it was 41.15% for school-age children aged six to 20 years [23]. In another study in the same country, the overall prevalence of caries was 52.0% in China [31]. Similar findings were observed in a study of 730 s- to fourth-grade children. Among the participants, 53.7% and 14.4% had caries in the primary and permanent dentition, respectively [18]. Moreover, a study of data from eight- to 12-year-old Brazilian children showed that the prevalence of dental caries was only 32.4% [32].

Although the prevalence of caries is still considered high among Saudi schoolchildren, this percentage is significantly lower than that reported in previous studies within the same community. This improvement is likely attributed to various enhancements and changes implemented in the last two years following the introduction of the 2030 Vision by the MOH school health program. The contributing factors include staff training courses, educational workshops for health councils in schools, collaborative efforts of the Ministry of Education and MOH, and awareness health campaigns conducted in schools and on social media channels. However, further studies are necessary to elucidate these positive changes.

This study can assist policymakers and public health officials in developing targeted interventions to address oral health inequalities among schoolchildren from different socioeconomic backgrounds. Furthermore, it will aid them in determining which school types require more attention in terms of oral health initiatives.

The interpretation of the current study results should consider specific limitations. The data utilised in the study were provided by the Saudi MOH, introducing uncertainties in the procedures for examination and data collection. Moreover, the datasets lack information on crucial factors such as family income, parental education, dietary habits, oral hygiene, and details regarding the number of examiners involved in the initiative and whether the examiners adhered to the initiative protocol. Furthermore, the available data do not provide clarity on whether the school visit was an initial assessment or a follow-up visit during the examination, which holds significance. Follow-up visits may have been influenced by the awareness programme and fluoride application, potentially impacting our study results.

Moreover, there are some challenges faced by the screening team. Firstly, since the team requested to examine all children in certain grades, absenteeism emerged as a significant obstacle for the screening team. Additionally, due to the large target number, the screening teams had to visit the school multiple times, leading to concerns from school authorities about the program interfering with their regular academic calendar. Moreover, a shortage of dentists involved in this initiative further exacerbated the situation. These issues collectively impacted the data collection process.

## Conclusions

In conclusion, this research revealed a notable prevalence of dental caries among first- and fourth-grade primary schoolchildren in SA, with approximately 42% affected. This study revealed that dental caries is more prevalent in public schools than in private schools and that there is a greater prevalence of dental caries in girls than in boys. Additionally, there is an age-related increase in dental caries, with fourth-grade students showing a greater prevalence of dental caries than their first-grade counterparts. To improve children’s oral health in SA, several recommendations have been proposed. These include implementing a well-organized oral health education program, integrating oral health information into school educational materials, and maintaining and improving school dental health programs with a focus on preventive measures. Prioritizing preventive services at an early age, conducting regular examination programs, and providing oral health care services to all children, especially those from public schools with lower socioeconomic backgrounds, are crucial steps. Furthermore, the development of policies to regulate school diets and close observation to minimize daily consumption of sugar-containing foods by students is emphasized. Further research is needed to explore the utilization of school type as an alternative measure of socioeconomic status in epidemiological dental caries surveys when individual data collection is challenging in Saudi society.

## Data Availability

The data that support the findings of this study are available from Saudi MOH, but restrictions apply to the availability of these data, which were used under license for the current study, and so are not publicly available. Data are however available from the authors upon reasonable request and with permission of Saudi MOH.
